# Understanding the Relationship between Sport Courage and Female Soccer Performance Variables

**DOI:** 10.3390/ijerph19084654

**Published:** 2022-04-12

**Authors:** Erkut Konter, Adam Gledhill, Yee Cheng Kueh, Garry Kuan

**Affiliations:** 1 Physical Education and Sports High School, İstanbul Gelişim University, Istanbul 34315, Turkey; erkut.konter@gmail.com; 2 Carnegie School of Sport, Leeds Beckett University, Leeds LS1 3HE, UK; adam.gledhill@leedsbeckett.ac.uk; 3 Biostatistics and Research Methodology Unit, School of Medical Sciences, Universiti Sains Malaysia, Kubang Kerian 16150, Malaysia; 4 Exercise and Sports Science Programme, School of Health Sciences, Universiti Sains Malaysia, Kubang Kerian 16150, Malaysia

**Keywords:** soccer, mastery, injury, level of participation, psychosocial factors

## Abstract

The purpose of this study was to examine the relationships between female soccer players’ courage and key performance variables (level of participation, injury past, being selected or non-selected by a national team, being starter or substitute). Methods: The Sport Courage Scale-31, by Konter and Ng (2012) and key performance variables were collected from 210 female soccer players aged 12 to 27 (M = 17.97 ± 3.34 years old). Spearman correlations and Mann–Whitney U tests were used to analyse the collected data. Results: The correlations between mastery (*r* = 0.196), determination (*p* = 0.239), assertiveness (*r* = 0.325), sacrifice behaviour (*r* = 0.182), total sport courage (*r* = 0.265) and age of female soccer players were found to be significant (*p* < 0.05). Female soccer players who have sustained an injury in the past scored significantly higher on the venturesome scale (*p* = 0.006) than those who have not sustained an injury in the past. In comparison, female soccer players who have not sustained an injury in the past or who have not been substituted had significantly more mastery than female soccer players who have sustained an injury in the past or who have been substituted (*p* = 0.017, *p* = 0.002, respectively). Conclusions: This study indicates that sport courage is related to key performance variables among female soccer players. Mastery and age seem to be related to courageous behaviour, whereas increasing venturesomeness might cause injuries in female soccer. Some relevant implications for practitioners can be drawn from the present findings.

## 1. Introduction

Consider how frequently a soccer coach, player, or fan has expressed the desire for their teams to be more courageous or assertive, especially during competitive performance. However, the relationship between sport courage and key performance variables is still unknown in most sports, including for female soccer [1]. The Multidimensional Courage Model is a model that has attempted to expand our understanding on sports courage and its association with key performance variables [2].

In the Model of Multidimensional Courage, Konter [2] defined sport courage as “a natural and developed, interactional and perceptual concept between person and situation, and the task at hand that enables person to move in mastery (self-confidence), determination, assertiveness, venturesome (coping with fear), and altruistic (self-sacrificed) behaviour on a voluntary basis in dangerous or difficult circumstances” (p. 966).

Multidimensional Interactional Sport Courage Models were suggested (Model 1 and Model 2) [2]. “Model-1” has six related parts from micro/particular to the macro/general levels. All the levels and parts in the Model-1 are related to one other. Firstly, the general model of sport courage (see Figure 1 in Konter [2]) includes different and related categories of courage, for example: personality traits, epistemological categories of courage, evolutionary and developmental processes of courage, social psychological dimension of courage, and interacting personal (age, gender, experience, achievement motivation, concentration, etc.) and situational factors (danger, risk, difficulty and fear at present) of courage. Model-2 illustrates the interactional nature of sport courage including the main factors, such as: type of sport, skill and task at hand, personal factors, situational factors and perception of the individual athlete. Model-1 can be used as a general model of understanding sport courage. Model-2 predicts the sport courage more specifically and interactionally. Researchers can concentrate on more specific models of sport courage from the proposed models [2].

To help guide future research, Konter and Beckman [1] reviewed the courage research in soccer and concluded that courage is frequently regarded as a significant factor in sport performance. They approached sport courage from a self-regulation standpoint, indicating that courage in soccer should enable players to take initiative and persist in goal pursuit despite the risk.

The Multidimensional Courage Model has influenced the increase in sport courage research in recent years. Konter’s [3] research comparing male and female adolescent students found four significant findings regarding sports courage. These included the following: (1) males have significantly higher points of mastery, determination, assertiveness, venturesome, and total sport courage (i.e., total SCS-31 scores) than females; (2) female sport participants who participate at least three times a week before the age of ten have significantly more points of determination and assertiveness than male and female non-sport participants; (3) male and female school team participants have significantly more points of sacrifice behaviours than males and females from non-school team participants; (4) males and females with very high levels of success perception in the motor domain (including physical education and sports) have significantly higher mastery, determination, assertiveness, venturesomeness, sacrifice behaviour, and total sport courage than males and females with good to low levels of success perception in the motor domain.

There is a lack of literature demonstrating the Multidimensional Courage Model can help to better understand an individual’s level of sport courage and various performance characteristics. As such, if one wishes to understand the relationship between sport courage and female soccer performance variables, it would be appropriate to investigate the concept of sport courage in this manner within female soccer.

Psychosocial factors could have influence on female soccer performance. The global expansion of female soccer has resulted in an increase in scholarly attention to psychosocial factors affecting performance and career development in female soccer; however, female soccer players are still disproportionately underrepresented in soccer psychology research [4]. Two qualitative studies [5,6] sought to advance this field of study by identifying psychosocial factors such as self-regulation, volitional behaviours, and relationships with peers, parents, and coaches as affecting female soccer players’ performance and career progression. Despite this understanding, these qualitative studies focus exclusively on female soccer in the United Kingdom (UK). As a result, findings may not be applicable outside of female soccer in the UK, and additional research into the psychosocial factors influencing female soccer performance is warranted. Additionally, it is worth mentioning that this UK study did not include sport courage as a variable. This may seem surprising, given the anecdotal and empirical evidence that sport courage is a desirable performance characteristic in athletes, e.g., [1,7,8]. This is indicative of a broader dearth of empirical evidence in female soccer examining the relationship between sport courage and performance variables [1,2,9]. Therefore, if we wish to promote sport courage as a desirable psychosocial characteristic among female soccer players, we must first investigate its relationship to various performance variables.

In summary, the study’s rationale is predicated on four key elements: (1) acknowledging the dearth of research into the psychology of female soccer [4]; (2) recognize risks that may be unique to female soccer players (e.g., increased injury risk; slower career progression). For instance, women had higher rates of ankle sprains, and more serious injuries of anterior cruciate ligament then men did [10], (3) a lack of understanding of the relationship between courage and performance variables in female soccer [1]; and (4) promote sport courage as a desirable psychosocial characteristic [1,7]. Informed by these four factors, the purpose of this study was to determine the relationship between female soccer players’ sport courage and performance variables (level of participation, national team participation, injury history, and team selection). By pursuing this purpose, we will be able to generate new knowledge about sport courage that can be used to inform both practical and theoretical recommendations, as well as contribute to the increased representation of female soccer players in soccer psychology research.

## 2. Materials and Methods

### 2.1. Participants

Following ethical approval and consent from gatekeepers, data were collected from 210 Turkish female soccer players aged 12 to 27 (*n* = 210, *M* = 17.97 ± 3.34 years, 196 amateur, 12 professional and 2 unstated). Table 1 discloses the descriptive information of the sample.

### 2.2. Measure

The Sport Courage Scale [9] and a personal information form were used to collect data of sociodemographic and key performance variables.

The Sport Courage Scale (SCS-31) has five subscales (mastery, determination, assertiveness, venturesome and sacrifice behaviour). The SCS-31 was initially developed and validated using a large sample (*n* = 768) of male and female athletes aged 13–22 years from a variety of team and individual sports. It was found to have a strong factor structure, validity, and reliability [9]. For the SCS-31, exploratory factor analysis and confirmatory factor analysis results indicated a good fit: comparative fit index = 0.93, Tucker–Lewis index = 0.93, root mean square error of approximation = 0.03, standardized root mean square residual = 0.06. Cronbach alpha coefficients for scale scores were as follows: determination = 0.82, mastery = 0.82, assertiveness = 0.72, venturesomeness = 0.72, sacrifice behaviour = 0.61. In addition, test–retest reliability of scale scores was established using 75 athletes’ responses: determination = 0.73; mastery = 0.77; assertiveness = 0.67; venturesomeness = 0.74; sacrifice behaviour = 0.62; total SCS-31 = 0.82 [9].

Each item of the SCS-31 was scored using five-point Likert-scale from 1 (strongly disagree) to 5 (strongly agree). Mastery related items of SCS-31 includes reversed items such as “My doubts regarding my abilities prevent me from succeeding in my sport”. Determination-related items of SCS-31 incorporate items such as “I perform to the best of my ability no matter how negative the current conditions are in my sport”. Assertiveness-related items of SCS-31 consist of items such as “I like to take the initiative in the face of difficulties in my sport”. Venturesome-related items of SCS-31 include items such as “I risk injury in order not to lose in my sport” and sacrifice-behaviour-related items of SCS-31 includes “I do not hesitate to compete, even when facing the possibility of defeat in my sport”.

The personal information form contained performance variables such as level of participation (How involved are you in soccer? (a) Professional, (b) Amateur), previous injury (Have you ever sustained a soccer injury that lasted longer than a week? (a) Yes, (b) No), national team selection and non-selection (How many times have you been selected for the national team?) with open and closed ended questions, and team selection whether as starter or substitute national team.

### 2.3. Procedure

The data were gathered through a convenience sampling technique with voluntary participation. The researchers obtained ethical approval from a local institute’s ethical committee prior to data collection. The first author then contacted the coaches of targeted teams via email and phone calls and briefed them on the research’s purpose, confidentiality, and anonymity of their participation. Following agreements, we established a date for data collection. The data collection period was May to November 2019. Soccer players received forms at their soccer training facilities. Participants were encouraged to answer honestly and ensured confidentiality. Two research assistants assisted participants aged 12 to 18 years old in clarifying the questions. Moreover, parental consent and assent forms were required for participants under 18 years old. Participants responded to these questions by filling in the blank space (for each item with their experiences). The collection of data took about 20 min to complete the set of forms.

### 2.4. Data Analyses

SPSS version 27.0 (IBM, New York, NY, USA) was used to conduct the data analysis. Normality of the numerical variables were checked by using histogram, Kolmogorov–Smirnov and Shapiro–Wilk tests. In overall, the numerical data was not normally distributed. Spearman correlations were used to analyse the collected data because it is not normally distributed. Additionally, Mann–Whitney U analysis was used as non-parametric test to compare mastery, determination, assertiveness, venturesomeness, sacrifice behaviour and total sport courage between level of play, injury history, national team participation, and team selection. The effect size was computed by using formula of z/√n, where z is the statistic value and n is the sample size. Effect size of 0.1, 0.3 and 0.5 are considered small, moderate and large, respectively [11]. The significance level was set at *p* < 0.05.

## 3. Results

The following subsections present the findings: age, level of soccer participation, injury history, national team participation, starters and substitutes, in accordance with the study’s objectives.

### 3.1. Sports Courage Scale (SCS-31) and Age

Spearman correlation analyses of the SCS-31 and age variables revealed significant correlations between the SCS-31 factors and age (Table 2).

### 3.2. Sports Courage Scale (SCS-31) and Level of Play

The results in Table 3 indicated that there was no significant relationship (*p* > 0.05) between SCS-31 and level of play (amateur and professional Soccer Participation).

### 3.3. Sports Courage Scale (SCS-31) and Injury History

Female soccer players with a history of injury had significantly higher venturesome scores (*p* = 0.006) than female soccer players without past injury. It also displayed the highest effect size compared with other courages. However, female soccer players who had not sustained an injury in the past had significantly more mastery points (*p* = 0.017) than female soccer players who had sustained an injury in the past (Table 4).

### 3.4. Sports Courage Scale (SCS-31) and Participation in National Team

There was no significant difference in SCS-31 and its factors between selected and non-selected national female soccer players (*p* > 0.05). Selected national female soccer players demonstrated higher determination (Mean Rank = 120.26 versus 100.63) and assertiveness (Mean Rank = 120.28 versus 100.63) points than non-selected national female soccer players (Table 5). However, there were not statistically significant.

### 3.5. Sports Courage Scale (SCS-31) and Team Selection

Female soccer players who were part of main team had significantly more mastery than substitute female soccer players (*p* = 0.002) (Table 6).

## 4. Discussion

The purpose of this study was to examine the relationships between measures of sport courage and performance variables in female soccer players. The results indicated that assertiveness, sacrifice behaviour, mastery, determination and total sport courage levels increased with age among female soccer players. Higher venturesomeness levels may play a significant role in the presence of injuries requiring a week or more in female soccer; higher mastery levels are associated with fewer injuries requiring a week or more in female soccer; and higher mastery levels are associated with being selected to start more frequently. These findings may provide potentially valuable information for stakeholders (e.g., players, coaches, sports medicine staffs, and sport psychologist practitioners) regarding the relative value of various indices of sport courage, their effect on playing level, and an understanding of their role in clinically meaningful injury risk reduction for injuries requiring one week or more of time-loss.

The discussion is divided into subsections on age, level of soccer participation, injury history, national team participation, and being a starter or substitute in relation to sports courage.

### 4.1. Sports Courage and Age 

The findings indicate that: (1) mastery, determination, assertiveness, sacrifice behaviour, and the total sport courage all have significant low positive correlations with age. Spearman correlation coefficients range from 0.182 to 0.325. This finding corroborates Konter’s [12] preliminary research findings. In female soccer, it appears that courage factors increase with age from 12 to 27 (M = 17.97 ± 3.34 years old). This could be due to the experience and the skill level increase with age, not the reverse. Additional research may be necessary to control for experience and skill level in order to obtain more conclusive results regarding the relationship between SCS-31 and age, as age increases with experience and skill level.

### 4.2. Sports Courage and Soccer Level of Participation

The results indicated that mastery, determination, assertiveness, sacrifice behaviour, and the total sport courage all decreased as the level of female soccer participation increased from amateur to professional. To a point, amateur female players have a higher assertiveness, a higher sacrifice behaviour, and a higher Total sport courage than professional female players. Professionalism in female soccer may entail developing more skills and playing safely, avoiding injury, and sticking to the tactics and performance roles rather than taking risks by being more assertiveness and exhibiting sacrifice behaviour. According to Konter [13], non-professional female soccer players had more sacrifice behaviour points than professional female soccer players. Additionally, Konter [14] discovered comparable findings regarding sacrifice behaviour in male soccer. Additional research is required to reach more convincing conclusions.

### 4.3. Sports Courage and Injury History

Venturesomeness (*p* = 0.006) was associated with an increased risk of sustaining an injury lasting more than one week, whereas mastery (*p* = 0.017) was associated with a lower risk of injury. Konter [15] demonstrated that athletes without a history of bone fracture have a significantly higher point of mastery than athletes with a history of bone fracture. However, the results with determination, assertiveness, venturesome, and total sport courage are inverted (participants’ mean age was 15.20 ± 1.56 years old; 118 males, 99 females). In addition, Konter [16] discovered that athletes with a history of broken bones had higher mastery, determination, assertiveness, venturesome, and total sport courage scores than athletes without a history of broken bones (participants’ mean age was 21.50 ± 2.31 years old; 56 males, 117 female). In general, it appears to corroborate findings concerning SCS-31 and injury history. Different outcomes could be attributed to different participants, including males and athletes from various sports. Nonetheless, the presence of mastery may reduce the likelihood of a female soccer player becoming injured; however, the presence of determination, venturesome, and sacrifice behaviour may result in increased injury. Although courage is necessary for soccer performance, it may also result in increased injury risk and prevent the female player from participating in soccer. Based on these findings, practitioners may find it beneficial to assess sport courage in female soccer players in order to assess the risk of injury. Understanding sacrifice behaviour levels in particular may enable targeted intervention by improving their courageous to reduce the risk of injury in female athletes.

The results of this section are constrained by the use of a single self-reported injury involving a week or greater time-loss. This was chosen for participant convenience, to minimise the risk of recall bias affecting the accuracy of the data collected, and because the majority of fixture schedules is weekly. Thus, understanding injuries that result in a week or more of lost time enables an assessment of the minimal impact that sport courage may have on player availability due to injury risk. This is significant because research from men’s professional soccer indicates that increased player availability correlates with improved performance outcomes in soccer [17,18]. Simply put, having a larger pool of players increases the chances of winning more games and competitions.

### 4.4. Sports Courage and National Team Participation

Descriptive statistic revealed that selected national female soccer players had higher mastery (mean rank: 117.84 vs. 101.22) and assertiveness (mean rank: 120.28 vs. 100.63) points than non-selected national female soccer players. Although the difference was not statistically significant and effect size was small, it is worth noting that this courage has important implications for athletes. Courage related to mastery and assertiveness appears to be critical for national team selection [19]. Female soccer players should be given more opportunities to improve their mastery and assertiveness in order to advance in their careers. They may require additional patience, practice sessions focused on mastery and assertiveness, successful outcomes, vicarious experiences, modelling, verbal preservations and encouragements [20,21], arousal management and self-regulation techniques, and psychological skills training [22], depending on their specific needs [1]. Additional research is necessary to draw firmer conclusions about courage, national team selection, and participation in female soccer at various levels.

### 4.5. Sports Courage and Being a Starter or Substitute

Female soccer players who start had significantly more mastery points than female soccer players who was substituted (*p* = 0.002). According to research, mastery’s relationship with self-confidence is critical to performance and psychological abilities [1,20,21,23,24].

### 4.6. Strengths, Weaknesses, and Future Directions for Research

This is the first study of its kind that examines the relationship between sport courage and various performance variables in female soccer. As such, it has generated new knowledge and responded to calls to advance the body of work on female soccer psychology. Notably, we discovered that sport courage indices are associated with an increased risk of injury in female Turkish soccer players. Thus, we recommend that coaches instill higher levels of sport courage in their soccer players in order to reduce the risk of injury in sports. This study was exploratory, retrospective, and cross-sectional in nature. As such, we have framed our assumptions and findings in terms of correlations and associations, rather than inferring causality. Prospective, longitudinal, multi-wave data collection and prospective tracking of players’ performance would bolster our claims. We acknowledge several limitations in the study such as small effect size on the differences on courage’s subscales between comparison groups. Moreover, sample size was also small for professional soccer players and possible confounding factors were not considered in the study. Thus, future research should include more professional players and identify possible confounding variables for sport courage. Future research may also benefit from examining interventions with female soccer players aimed at developing potentially more facilitative dimensions of sport courage, such as mastery, while also assisting athletes in modulating other dimensions of sport courage, such as venturesome and sacrifice behaviour, that may have a more detrimental effect on specific performance aspects. In their narrative review, Milanović and colleagues explained that better understanding of physiological and physical demands of female soccer would lead to better criteria for future players development and success [25]. Hence, association between courage and injury risk factors based on physical or physiological variables are also suggested for future investigation.

## 5. Conclusions

To conclude, mastery appears to have the greatest facilitative potential for female soccer players in Turkey when compared with the other dimensions of sport courage. It is associated with being a team starter, being selected for a national team, and having a lower risk of injury for injuries requiring a week or more of recovery time. Mastery and age seem to be importantly related to courageous behavior, whereas increasing venturesomeness can cause injury in female soccer players. These could be important implications for practitioners who may find it beneficial to examine sport courage in female soccer when evaluating the risk of injury. Sport practitioners may be able to provide appropriate intervention by improving mastery level among female athletes and thus reduce risk of injury. The study brings light that sport courage is related to the key performance variables among female soccer players.

## Figures and Tables

**Table 1:** Descriptive characteristics of participants.

Variables	Mean (SD)	*n* (%)
**Age**	17.97 ± 3.34	209 (99.5)
Unstated		1 (0.05)
**Performance Variables**		
Level of Soccer		208 (99.0)
Unstated		2 (1.0)
Injury status		204 (97.1)
Unstated		6 (2.9)
Nat.Team Participation		208(99.0)
Unstated		2 (1.0)
Starter or Subs		205 (97.6)
Unstated		5 (2.4)
**Soccer Courage Factors**		
Mastery	23.85 ± 6.40	210 (100)
Determination	34.12 ± 9.65	210 (100)
Assertiveness	25.73 ± 6.11	210 (100)
Venturesomeness	14.67 ± 4.62	210 (100)
Sacrifice behaviour	14.72 ± 4.26	210 (100)
Total Sport Courage	113.09 ± 25.05	210 (100)

**Table 2:** Spearman correlations of the Sports Courage Scale and age of the participants.

Variables	Mastery	Determination	Assertiveness	Venturesome	Sacrifice Behaviour	Total Sport Courage
Age	0.196 *	0.239 *	0.325 *	0.075	0.182 *	0.265 *

* p < 0.05.

**Table 3:** Results of the Sports Courage Scale and level of play.

Variables	Groups	Mean Rank	Z-Statistics	*p*-Value	Effect Size
Mastery	Level of play				
	1-Amateur	104.13	−0.022	0.982	<0.001
	2-Professional	104.52			
Determination	Level of play				
	1-Amateur	92.96	−0.685	0.493	0.002
	2-Professional	105.21			
Assertiveness	Level of play				
	1-Amateur	93	−0.683	0.495	0.002
	2-Professional	105.2			
Venturesome	Level of play				
	1-Amateur	93.38	−0.662	0.508	0.002
	2-Professional	105.18			
Sacrifice behaviour	Level of play				
	1-Amateur	79.17	−1.508	0.132	0.011
	2-Professional	106.05			

**Table 4:** Results of the Sports Courage Scale and previous injury.

Variables	Groups	Mean Rank	Z-Statistics	*p*-Value	Effect Size
Mastery	Injury Past				
	1-Yes	87.26	−2.383	0.017 *	0.028
	2-No	108.85			
Determination	Injury Past				
	1-Yes	111.89	−1.468	0.142	0.011
	2-No	98.59			
Assertiveness	Injury Past				
	1-Yes	110.49	−1.25	0.211	0.008
	2-No	99.17			
Venturesome	Injury Past				
	1-Yes	120.12	−2.762	0.006 *	0.038
	2-No	95.16			
Sacrifice behaviour	Injury Past				
	1-Yes	114.40	−1.866	0.062	0.017
	2-No	97.54			

Note. Mann–Whitney U Tests applied. * p < 0.05.

**Table 5:** Results of Sports Courage Scale and national team participation.

Variables	Groups	Mean Rank	Z-Statistics	*p*-Value	Effect Size
Mastery	Nat.Team Part.				
	(a) National	117.84	−1.586	0.113	0.012
	(b) Non-national	101.22			
Determination	Nat.Team Part.				
	(a) National	120.26	−1.877	0.061	0.017
	(b) Non-national	100.63			
Assertiveness	Nat.Team Part.				
	(a) National	120.28	−1.877	0.061	0.017
	(b) Non-national	100.63			
Venturesome	Nat.Team Part.				
	(a) National	98.45	−0.721	0.471	0.003
	(b) Non-national	105.99			
Sacrifice behaviour	Nat.Team Part.				
	(a) National	104.99	−0.032	0.974	<0.001
	(b) Non-national	104.57			
Mastery	Nat.Team Part.				

Note. Mann–Whitney U Tests applied; Nat.Team Part.: National team participation.

**Table 6:** Results of Sports Courage Scale and team selection.

Variables	Groups	Mean Rank	Z-Statistics	*p*-Value	Effect Size
Mastery	Starter-Subs				
	1-Starter	111.66	−3.07	0.002 *	0.046
	2-Subs	84.35			
	Nat.Team				
Determination	Starter-Subs				
	1-Starter	104.57	−0.557	0.577	0.002
	2-Subs	99.62			
	Nat.Team				
Assertiveness	Starter-Subs				
	1-Starter	193.6	−0.214	0.830	<0.001
	2-Subs	101.7			
	Nat.Team				
Venturesome	Starter-Subs				
	1-Starter	102.04	−0.34	0.734	<0.001
	2-Subs	105.06			
	Nat.Team				
Sacrifice behaviour	Starter-Subs				
	1-Starter	101.08	−0.683	0.494	0.002
	2-Subs	107.14			
	Nat.Team				

Note. Mann–Whitney U Tests applied, * p < 0.05, Subs Nat.Team: Substituted national team.

## Data Availability

The data are available upon request from the authors.
